# Restoring degraded soils: The transformative impact of Fanya Juu terraces on soil characteristics in the erosion-prone Magera watershed, Southern Ethiopia

**DOI:** 10.1371/journal.pone.0342826

**Published:** 2026-02-19

**Authors:** Fekadu Fanjana Falta, Selemon Thomas Fakana, Nega Kesete Kassie

**Affiliations:** 1 Department of Natural Resources Management, College of Agriculture, Wolaita Sodo University, Wolaita Sodo, Ethiopia; 2 State Key Laboratory of Soil and Water Conservation and Desertification Control, College of Soil and Water Conservation Science and Engineering (Institute of Soil and Water Conservation), Northwest A&F University, Yangling, China; 3 Department of Environmental Science, College of Agriculture and Natural Resources, Gambella University, Gambella, Ethiopia; 4 Department of Natural Resources Management, College of Agriculture and Natural Resources, Mekdela Amba University, Wollo, Ethiopia; Rodale Institute, UNITED STATES OF AMERICA

## Abstract

Excess runoff-induced soil erosion significantly degrades farmland fertility, quality, agricultural productivity, and long-term sustainability. Although Fanya Juu terraces are widely implemented for mitigating these effects, previous studies rarely examined how slope position and conservation age influence soil recovery, as they focused on broad effectiveness. This study addressed the gap by analyzing soil characteristics across slope position and Fanya Juu structures of 5 and 10 years, compared with non-conserved land, to provide nuanced guidance for implementation. Soil samples (0–20 cm depth) were collected from 27 plots (3 slope positions x 3 treatments x 3 replications) in the watershed during the fall season of 2024. Normality and homogeneity of variance were assessed prior to analysis using the Kolmogorov-Smirnov and Levene’s tests, respectively. A two-way ANOVA and Tukey’s Least Significant Difference (LSD) test at a 5% probability level was used to analyze the data. The result revealed significant improvements with conservation age and slope position. Over ten years, Fanya Juu terraces have increased organic carbon (28%), total nitrogen (66%), available phosphorus (43%), CEC (39%), soil moisture (14%), silt (44%), and aggregate stability (51%) while reducing bulk density by 19%. The findings highlight that the Fanya Juu effectiveness depends strongly on slope and structure age, providing quantitative evidence for optimizing terrace design and long-term soil and water conservation planning in erosion-prone farmlands.

## 1. Introduction

Soil erosion driven by excess surface runoff poses a major global environmental challenge, leading to depletion of fertile topsoil [1,2], reduced agricultural productivity, and degradation of ecosystem services [3,4], and is a hindrance to increased yields of crops and sustainable agricultural land management [5–8]. Agricultural expansion through the conversion of forests and grasslands contributes to nearly 80% of global soil erosion, resulting in severe on and off-site impacts on water resources, biodiversity, carbon sequestration, and air quality [9–12]. In sub-Saharan Africa, soil erosion results in lower agricultural productivity [13,14].

Ethiopia is profoundly affected by runoff-induced soil erosion, which has negatively impacted farmland productivity [15,16]. According to [17], the national average loss of approximately 30 t ha^-1^y^-1^ exceeds an acceptable threshold. In the 1970s and 1980s, Ethiopia implemented soil and water conservation practices to rehabilitate degraded land, reduce erosion, and restore soil fertility for improved agricultural productivity [18–20]. Fanya Juu practices are widely adopted and maintained to demonstrate tangible benefits to farmers, such as increased crop yields, improved household income [21], and enhanced resilience to climate variability [22]. Terraces, Fanya Juu, cut-off drains, bunds, and grassed waterways have been widely adopted through community engagement and incentive-driven programs [20,23]. The Fanya Juu technique involves excavating a trench approximately 0.5 meters in depth and 0.5 meters in width along the contour. Concurrently, an embankment is formed on the upslope side of the trench, serving as an initial line of defense against surface runoff and soil erosion [24] in diverse agroecological zones and areas prone to erosion [25]. Its efficacy is contingent upon the prevailing agroecological conditions and the particular conservation practices employed [26,27].

Despite these, the effectiveness of soil and water conservation practices remains inconclusive within the existing literature [3,4,28]. Several studies report improvements in soil organic carbon, nutrient availability, and water retention in conserved lands [26,29–32]. Soil and water conservation practices, such as Fanya Juu, have been extensively recognized for their efficacy and ability to mitigate soil erosion [9,33–35]. Other studies found negligible soil texture, bulk density, and slope stabilization even after decades of implementation, which is linked to slope position, agroecological management, and maintenance practices [9]. Furthermore, most of the existing studies are short-term or cross-sectional, providing limited insights into the long-term impacts of Fanya Juu [9]. Besides, prior research has examined soil characteristics on soil and water conservation by considering variations over time. This body of work has integrated physical soil and water conservation measures with ‘desho’ grass, conducted comparative analyses of Fanya Juu against different SWC techniques, and explored the interaction of newly implemented Fanya Juu with various tillage operations across multiple soil types and agricultural methodologies [3,31,36,37].

Located in an area with high population pressure and a degraded Omo-Turkana basin, the Magera sub-watershed is characterized by low availability of water, sparse cover of vegetation with high patchiness, low soil moisture content, and the land of the area is severely degraded [38]. This region is a priority area for Ethiopia’s national land restoration efforts, including the large-scale Sustainable Land Management Program (SLMP) and the Green Legacy Initiative [36]. In the area, agricultural land productivity is jeopardized by soil erosion and degradation resulting from undulating topography and population pressure, leading to cultivation in marginal areas. In response to the significant and urgent challenges associated with land degradation, comprehensive watershed management strategies have been executed in critical areas within the Magera watershed by the initiative of the Sustainable Land Management Program (SLMP) project starting from 2013/14. Both biological and physical SWC works introduced under the SLMP project include Fanya Juu, soil and stone bunds, deep trenches, hillside terraces, water harvesting systems, and ponds on communal lands [38].

Even though the research employed a range of methodologies for assessing physical soil water conservation (SWC), information related to the influence of Fanya Juu on the characteristics of soils is limited in areas with high population pressure, previously severely degraded, and annual crop cultivation on erosion-prone lands. Comprehending the impact of SWC practices on soil characteristics across various agroecological and degradation regions is essential for making informed decisions regarding effective strategizing and execution. The current study aimed to evaluate the impact of Fanya Juu SWC measures on the soil physicochemical characteristics of agricultural land in areas susceptible to erosion.

## 2. Materials and methods

### 2.1 Description of the study area

Magera sub-watershed is found in Boloso Bombbe woreda with geographical coordinates 37.440–37.660 East and 7.030–7.190 North, and the elevation of the woreda is 501–2500 meters above sea level. The mean minimum and maximum temperatures are 12.6 and 25.0 ◦C.

**Fig 1:**
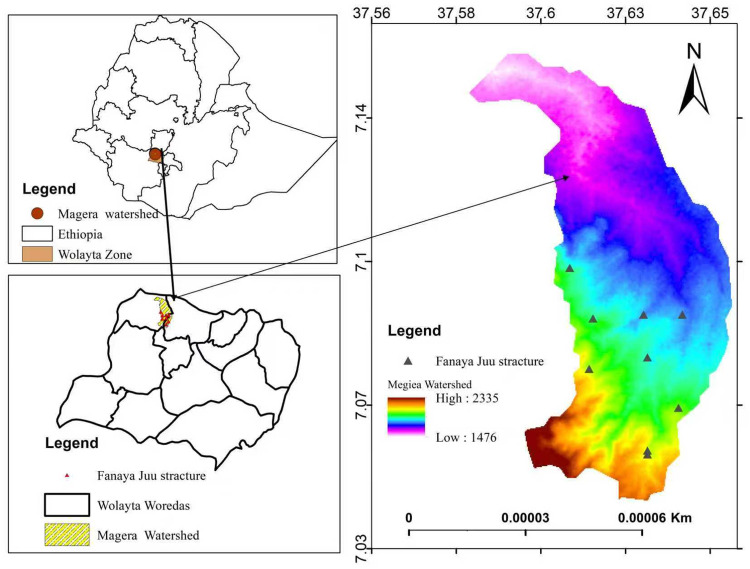
Location of magera watershed.

The area receives an average annual rainfall of 1400 mm. The rainfall occurs from March to early October, with the highest amounts in July and August (principal meteorology station at Areka), 26 kilometers away from the woreda [39]. The area encompasses various agroecological zones, including Kola, characterized by low elevation and high temperatures; Dega, defined by high elevation and lower temperatures; and Woyna Dega, representing medium elevation and temperature. The total area of the woreda accounts for 62.28%, 14.28%, and 23.44% of Kola, Dega, and Woyna Dega, respectively [39].From the total surface area of the woreda, 17.4% (47.1 km^2^) is occupied by the Magera watershed and drains south in the northern direction. The specific watershed elevation is 1502–2335 m above sea level and geographically located between 702’30’‘-707’41’‘ North and 36036’25’‘- 37039’15’‘ East (Fig 1). The sources of shapefiles are obtained from freely available resources for academic purposes in DIVA-GIS [40] and hydro shades [41].

### 2.2 Soil sampling and data collection

For this study, a field survey was conducted to assess the presence of adjacent lands featuring Fanya Juu structures that are 5 and 10 years old, as well as non-conserved lands with a comparable land-use history. This study employed a chronological sequence approach to evaluate the effects of soil and water conservation (SWC) practice duration. In 2024, the adjacent fields within the same agroecological zone with Fanya Juu SWC structures that were implemented at different times in the past. Specifically, the sites with 5 and 10-year Fanya Juu implementation were identified, and for control, sites with no SWC were used. The control 0-year sites (managed similarly but without SWC) served as a spatial proxy for the pre-implementation condition.

Fanya Juu, a Kiswahili term commonly used in East Africa meaning “throw it upwards,” refers to a physical soil and water conservation (SWC) technique. This method involves constructing a trench along a contour line, typically measuring approximately 0.5 meters in both depth and width. An embankment is then formed on the upslope side of the trench. This embankment serves as a primary barrier against surface runoff and soil erosion [3]. This study utilized a chrono sequence

**Fig 2:**
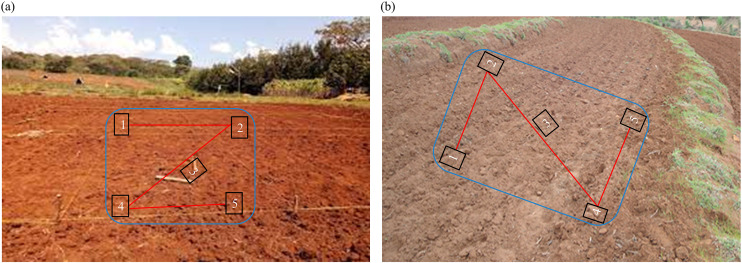
Soil sampling points: a) non-conserved land, and b) land with Fanya Juu.

Before collecting soil samples, the slopes of the farmland were recorded using a clinometer. Given that the Fanya Juu structures were implemented on slopes ranging from 3% to 30%, the area was categorized into three slope classes to mitigate variability in soil properties attributable to elevation differences: lower (3–10%), middle (10–20%), and upper (20–30%). For each treatment type, non-conserved farmland and farmland with 5-year and 10-year Fanya Juu structure transect lines were established across each slope class. In the non-conserved land-utilization type, three sample plots (15 m × 15 m) were randomly established along each transect line, and five soil pits were excavated in a ‘Z’ design from the corners, center, and the diagonal of every plot to prepare a composite soil sample (Fig 2).

For lands with Fanya Juu structures, soil samples were collected along the transect line from five pits (two from the upper section, two from the lower section, and one from the middle) situated between two successive structures, with three replications (3 plots for each, which are in different locations), and composite soil samples were prepared. All samples, totaling 27 composite soil samples, were collected from the top 0–20 cm depth and calculated as follows: 3 slope gradients (lower, middle, and upper slope classes) multiplied by 3 treatments (non-conserved, 5-year, and 10-year Fanya Juu conserved farmlands) multiplied by 3 replications of sample plots multiplied by 1 soil depth. The soil sampling depth of 0–20 cm was selected as it represents the standard plow layer in conventional agricultural systems for this region and is the established benchmark for assessing topsoil fertility, nutrient availability, and short-term organic matter dynamics that most directly influence crop growth [3]. Bulk density sampling was conducted using the core method with a core sampler from the same plots, allowing for the determination of soil moisture content (SMC). All the soil samples are collected at the end of the main rainy season and before the start of the dry season (late September to October). Following collection, soil samples were conveyed to the National Soil Testing Laboratory at Sodo, where soil air-drying and sieving were performed before laboratory analysis. The soil samples were subsequently analyzed for various parameters, including soil texture, bulk density (g/cm^3^), moisture content (%), pH, organic carbon (g kg^-1^), available phosphorus (g kg^-1^), exchangeable potassium (g kg^-1^), total nitrogen (g kg^-1^), and cation exchange capacity (cmol kg^-1^), by standard soil science procedures.

#### 2.2.1 Analysis of soil physical properties.

The hydrometer method was employed for particle size analysis [42], and the USDA soil textural triangle was utilized for soil texture classification [43]. The method outlined by [44] was used for determining the gravimetric soil moisture content (SMC (%)). Initial weights of soils were recorded before oven-drying the soil, which was subsequently dried for 24 hours at 105 °C and weighed again. The gravimetric moisture content of the soil was calculated using the following formula.


$$\text{MC}(\%)=\frac{\text{Wwet}-\text{Wdry}}{\text{Wdry}}$$ (1)

Where: MC is the moisture content of the soil, Wwet is the fresh weight of the soil, and Wdry is the weight after oven drying.

The core method was utilized to determine soil bulk density (BD) and calculated as the mass of oven-dried soil (105°C) divided by its volume [45].


$$\rho \left(\text{g}/\text{c}{\text{m}}^{\text{3}}\right)={\text{M}}_{\text{s}}/{\text{V}}_{\text{b}}$$ (2)

Where ρ = bulk density of soil (g/cm^3^), Ms = mass of over-dry soil (g), Vb = total volume of the soil (cm^3^).

The determination of soil structure stability index SSI (%) was derived from the results of soil lab analysis and given by the formula [46].


$$\text{SSI}(\%)=\frac{\text{1}.\text{724OC}\%}{\left(\text{silt }\%+\text{clay }\%\right)}*\text{1}\text{0}\text{0}$$ (3)

#### 2.2.2 Analysis of soil chemical properties.

Measuring soil pH was conducted by using a pH meter in a 1:2.5 soil: water ratio [47]. The wet digestion method by [44] was utilized to estimate the content of soil carbon. Available phosphorus was determined using the Olsen method [48,49]. The potassium (Exch. K) content was determined by the extraction method using one of the spectrometric determination methods [50]. Total nitrogen (TN) was analyzed using the Kjeldahl digestion, distillation, and titration method as described by [51]. A concentrated sulfuric acid solution is utilized to oxidize the organic matter. Soil Cation Exchange Capacity (CEC) was determined using the ammonium acetate saturation method at pH 8.2 [52].

### 2.3 Statistical analysis

The collected data were systematically organized, analyzed, and interpreted using Origin Pro software, which has robust capabilities for performing advanced statistical tests and generating high-quality graphs. In this study, the implementation duration (Control, 5-year, and 10-year) was treated as a categorical fixed effect in one-way ANOVA. Various physical and chemical soil characteristics served as dependent variables in the statistical analysis of soil qualities, while slope gradients and the age of the Fanya Juu terrace were used as independent variables. Normality and homogeneity of variance were assessed prior to analysis using the Kolmogorov-Smirnov and Levene’s tests, respectively. To evaluate Fanya Juu’s performance within specific slope classes, one-way analysis of variance was employed. A two-way ANOVA was conducted using OriginPro 2025 software to assess the effects of slope gradient and Fanya Juu’s age on soil properties. When significant differences were found, mean comparisons were performed using Tukey’s Least Significant Difference (LSD) at a 5% significance level. Claiming effects over time are thus based on significant differences between these categorical groups sampled in the same year. Additionally, the relationship between soil characteristics and the age of the Fanya Juu terrace was examined using a Pearson’s correlation matrix at a significance level of p < 0.05 and p < 0.01. This was to evaluate the strength and direction of linear relationships between continuous variables

## 3. Results and analysis

### 3.1 Influence of the Fanya Juu age and slope gradient on the physical characteristics of soil

**Fig 3:**
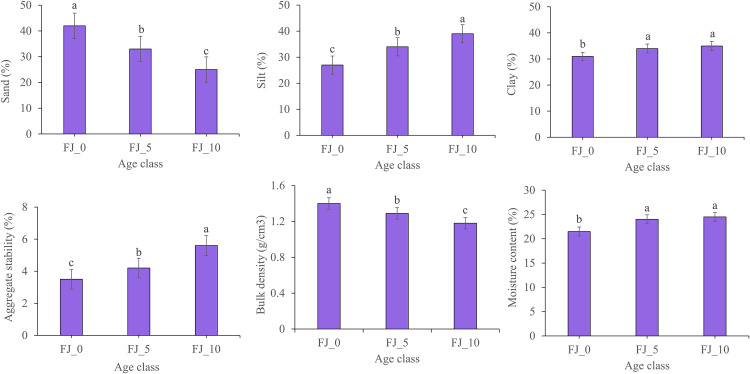
Soil physical properties (Sand, Silt, Clay, Moisture Content, Bulk Density, Aggregate Stability) as affected by the age of Fanya Juu terraces (0, 5, and 10 years). Different letters above bars indicate statistically significant differences (p < 0.05) based on Tukey’s LSD test.

Soil physicochemical characteristics are significantly influenced by the age of the Fanya Juu and the slope class of the area. With the Fanya Juu implementation age, sand, aggregate stability, and bulk density showed significant differences. Silt, clay, and moisture content showed significant differences with non-conservation implemented lands, but did not show differences between FJ_5 and FJ_10 (Fig 3).

**Fig 4:**
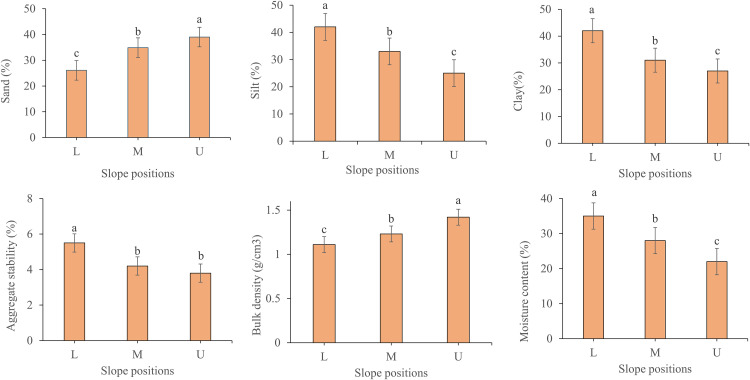
Soil physical properties (Sand, Silt, Clay, Moisture Content, Bulk Density, Aggregate Stability) as affected by the slope position (Lower, Middle, and Upper). Different letters above bars indicate statistically significant differences (p < 0.05) based on Tukey’s LSD test.

The influence of slope position significantly influenced the sand, silt, clay, bulk density, and moisture content of soils. However, soil aggregate stability was higher in the lower slope position (L) (Fig 4).

### 3.2 Influence of the Fanya Juu age and slope gradient on chemical properties of soil

**Fig 5:**
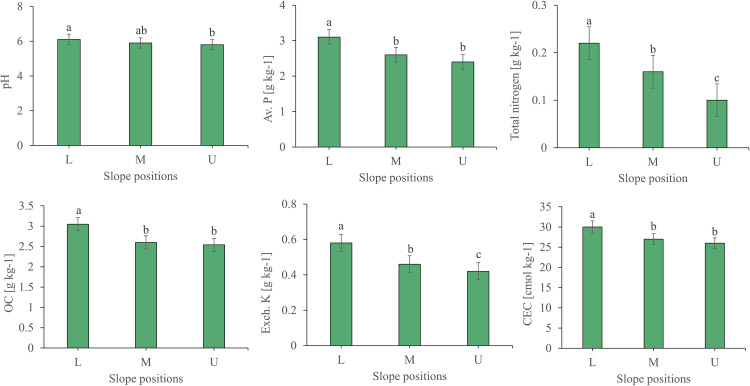
Soil chemical properties (pH, OC, Av. P, Total nitrogen, Ex. K, CEC) as affected by the age of Fanya Juu terraces (0, 5, and 10 years). Different letters above bars indicate statistically significant differences (p < 0.05) based on Tukey’s LSD test.

The soil’s chemical properties are significantly influenced by the age of Fanya Juu and the slope of the land. In the age class, available phosphorus (Av. P), Exchangeable Potassium (K^+^), total nitrogen (TN), organic carbon (OC), and Cation exchange capacity (CEC) showed significant differences. The result of pH does not show a significant difference (Fig 5).

**Fig 6:**
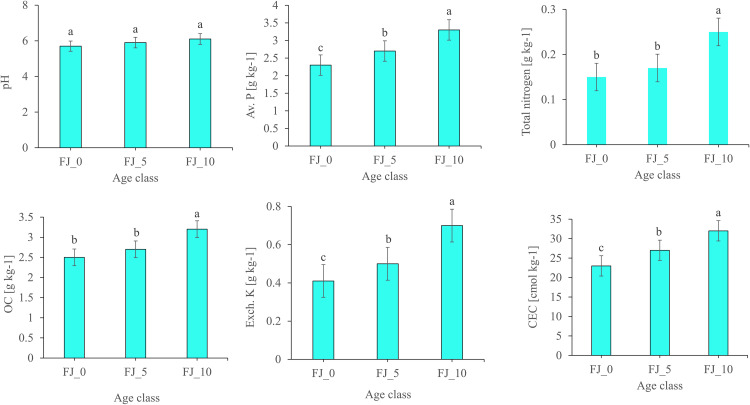
Soil chemical properties (pH, OC, Av. P, Total nitrogen, Ex. K, CEC) as affected by the slope position (Lower, Middle, and Upper). Different letters above bars indicate statistically significant differences (p < 0.05) based on Tukey’s LSD test.

The soil chemical properties showed significant variation in the lower slope position. As illustrated in (Fig 5), the pH, available Phosphorus, organic carbon, and CEC are higher in the lower position of the slope, and there are also significant differences in the medium and upper slope positions. The total nitrogen concentration at different slopes is significantly different in all positions. The exchangeable K didn’t show a significant difference (Fig 6).

### 3.3 Influence of the age of Fanya Juu on soil physicochemical properties at slope positions

As presented in Table 1, at the lower slope, with 10-year practices, soil and water conservation with Fanya Juu, Sand content, and bulk density decreased, and clay, silt, moisture content, and aggregate stability showed higher values. Within 5 and 10-year conservation, sand, silt, and clay didn’t significantly differ. The chemical properties of soil also showed great variation at different ages; organic carbon, available phosphorus, exchangeable potassium, total nitrogen, and cation exchange capacity showed significant differences. The pH of the soil was found to be significantly different in all age classes at lower slopes (Tables 1–2).

**Table 1:** Soil properties modified by the age of Fanya Juu at different slope positions.

Factors		Sand	Silt	Clay	MC	BD	AS	PH	OC	Av. P	K+	TN	CEC
Slope	Age	(%)	(%)	(%)	(%)	(g/cm^3^)	(%)		[g kg^−1^]	[g kg^−1^]	[gkg^−1^]	[gkg^−1^]	[cmolkg^−1^]
L	0	31.67 ± 4.62^a^	32.00 ± 2.00^a^	36.33 ± 3.05^a^	28.93 ± 2.20^a^	1.21 ± 0.08^a^	3.10 ± 2.00^a^	6.43 ± 0.03^a^	1.89 ± 0.12^a^	2.45 ± 0.08^a^	0.67 ± 0.02^a^	0.08 ± 0.02^a^	24.46 ± 2.20^a^
	5	21.33 ± 3.51^b^	36.67 ± 1.15^ab^	42.00 ± 4.36^b^	28.36 ± 2.28^a^	1.16 ± 0.03^ab^	5.20 ± 1.15^b^	6.81 ± 0.08^a^	2.60 ± 0.21^b^	3.24 ± 0.14^b^	0.75 ± 0.14^ab^	0.22 ± 0.04^b^	36.50 ± 4.46^b^
	10	18.80 ± 5.50^b^	39.00 ± 1.00^b^	43.20 ± 4.73^b^	34.88 ± 1.02^b^	1.12 ± 0.02^b^	5.90 ± 1.00^b^	6.37 ± 0.24^a^	3.16 ± 0.27^b^	3.41 ± 0.04^b^	0.84 ± 0.22^b^	0.24 ± 0.01^b^	39.60 ± 6.54^b^
M	0	35.67 ± 4.93^a^	28.00 ± 2.00^a^	36.33 ± 3.21^a^	23.28 ± 1.95^a^	1.30 ± 0.06^a^	3.60 ± 2.00^a^	5.13 ± 0.08^a^	2.19 ± 0.12^a^	2.23 ± 0.14^a^	0.51 ± 0.10^a^	0.12 ± 0.19^a^	26.46 ± 2.24^a^
	5	29.00 ± 3.00^b^	30.67 ± 1.52^a^	40.33 ± 2.88^b^	25.96 ± 3.03^ab^	1.17 ± 0.05^b^	4.07 ± 1.52^b^	5.22 ± 0.04^ab^	2.50 ± 0.21^ab^	2.70 ± 0.25^b^	0.55 ± 0.09^a^	0.18 ± 0.44^b^	29.60 ± 0.20^b^
	10	22.33 ± 4.5^c^	35.67 ± 3.05^b^	42.00 ± 1.73^b^	27.01 ± 1.81^b^	1.15 ± 0.05^b^	4.68 ± 3.05^b^	5.62 ± 0.21^b^	2.96 ± 0.27^b^	2.76 ± 0.22^b^	0.70 ± 0.07^b^	0.20 ± 0.01^b^	32.04 ± 0.70^c^
U	0	41.66 ± 2.51^a^	23.67 ± 1.52^a^	34.67 ± 1.15^a^	18.13 ± 0.66^a^	1.46 ± 0.40^a^	3.40 ± 1.52^a^	6.04 ± 0.03^a^	2.23 ± 0.01^a^	2.13 ± 0.04^a^	0.24 ± 0.012^a^	0.10 ± 0.00^a^	25.90 ± 3.91^a^
	5	38.00 ± 1.00^ab^	25.67 ± 0.57^a^	36.33 ± 0.57^ab^	23.14 ± 0.60^b^	1.32 ± 0.08^a^	3.77 ± 0.57^a^	5.60 ± 0.06^b^	2.43 ± 0.09^a^	2.32 ± 0.14^ab^	0.46 ± 0.03^ab^	0.11 ± 0.00^a^	26.90 ± 1.23^a^
	10	35.66 ± 1.52^b^	26.67 ± 1.15^b^	37.67 ± 0.57^b^	22.40 ± 1.34^b^	1.39 ± 0.05^b^	4.63 ± 1.15^b^	5.69 ± 0.15^b^	3.03 ± 0.27^b^	2.68 ± 0.25^b^	0.62 ± 0.05^b^	0.16 ± 0.07^b^	28.70 ± 1.32^b^

**Table 2:** Two-way analysis of variance for the Fanya Juu implementation age classes, slope class, and interaction effect on soil characteristics.

	Source of variation					
	Age classes		Slope classes		Age*Slope	
Soil properties	F	p	F	P	F	P
Sand	30.24	0.00	18.41	0.00	8.71	0.69
Silt	84.18^*^	0.00	21.18^*^	0.00	5.98	0.04
Clay	8.56^*^	0.00	7.78^*^	0.00	12.45	0.03
BD	5.76^*^	0.00	5.38^*^	0.00	6.59	0.02
MC	28.37^*^	0.00	9.69^*^	0.00	5.82	0.04
OC	52.38^*^	0.00	32.51^*^	0.00	2.81	0.03
TN	4.89^*^	0.00	94.16^*^	0.00	9.48	0.02
pH	94.22^*^	0.00	40.34^*^	0.00	8.51	0.02
Av. P	42.91^*^	0.00	38.62^*^	0.00	6.62	0.03
K^+^	39.87*	0.00	101.21^*^	0.00	7.89	0.02
CEC	32.43^*^	0.00	40.70^*^	0.00	3.1	0.04

*Significant at p < 0.05.

At the medium slope position, sand, silt, clay, moisture content, bulk density, and aggregate stability showed significant differences between non-conserved and 5- and 10-year-old Fanya Juu practices. Likewise, 5- and 10-year-old Fanya Juu practices did not show significant differences in clay, bulk density, and aggregate stability. With the application of Fanya Juu soil and water conservation measures for 10 years at medium slopes, the pH, organic carbon, and available phosphorus, exchangeable potassium, total nitrogen, and cation exchange capacity were found to be significantly different (Table 1).

At the upper slope position, sand, silt, and clay moisture content, bulk density, and aggregate stability showed significant differences as compared to non-conserved and 5- and 10-year-old Fanya Juu practice. On the other hand, 5- and 10-year-old Fanya Juu practices did not show significant differences in soil moisture content. The pH, organic carbon, available phosphorus, exchangeable potassium, total nitrogen, and cation exchange capacity were found to be significantly different at 10-Fanya Juu practice. On the contrary, at 5 years old, Fanya Juu, organic carbon, total nitrogen, and cation exchange capacity are not significantly different (Table 1). In addition to the significant main effects of slope and stand age on soil properties, their interaction was also significant, indicating that the influence of slope varied across different age classes (Table 2).

### 3.4 Pearson correlation of soil properties in different age classes

As presented in Table 3 below, the Fanya Juu soil and water conservation practice influenced soil physicochemical characteristics. The silt content positively correlated with all chemical soil properties under investigation. Clay and moisture content and aggregate stability index correlated positively with pH, organic carbon, available phosphorus, total nitrogen, and cation exchange capacity. In contrast, sand content and bulk density had negative correlations with exchangeable potassium and cation exchange capacity in non-conserved lands. Similar to non-conserved land, silt contents were strongly positively correlated with all soil chemical properties in lands with 5-year Fanya Juu practice. Besides, clay and moisture content are also positively correlated with pH, organic carbon, and available phosphorus. Silt content is strongly and positively correlated with soil chemical properties. On the contrary, sand content in 10-year Fanya Juu conserved land showed a strong negative correlation with organic carbon, cation exchange capacity, total nitrogen, and exchangeable potassium. In 10-year conserved soils, clay content was also strongly positively correlated with organic carbon, available phosphorus, exchangeable potassium, and total nitrogen.

**Table 3:** Correlation among soil physicochemical properties at three age classesclasses.

	Physical properties						
Age (yrs)	Chemical properties	Sand	Silt	Clay	BD	MC	AS
0	pH	−0.35	0.71*	0.88*	−0.48	0.92**	0.64
	OC	−0.56	0.75*	0.82*	−0.23	0.86*	0.75*
	Av. P	−0.46	0.94**	0.79*	−0.37	0.78*	0.45
	Ex. K	−0.71*	0.90**	0.64	−0.48	0.41	0.52
	TN	−0.67	0.80*	0.62	−0.43	0.89*	0.70*
	CEC	−0.78*	0.90*	0.83*	−0.49	0.80*	0.85*
5	pH	−0.52	0.74*	0.71*	−0.59	0.78*	0.40
	OC	−0.78*	0.92*	0.78*	−0.78*	0.90**	0.91**
	Av. P	−0.48	0.89*	0.86*	−0.54	0.71*	0.60
	Ex. K	−0.78*	0.91**	0.56	−0.52	0.49	0.40
	TN	−0.90**	0.90**	0.40	−0.43	0.79*	0.87*
	CEC	−0.87*	0.91**	0.81*	−0.41	0.83*	0.48
10	pH	−0.73*	0.80*	0.71*	−0.40	0.82*	0.62
	OC	−0.89*	0.77*	0.91**	−0.78*	0.75*	0.79*
	Av. P	−0.45	0.91*	0.71*	−0.43	0.72*	0.56
	Ex. K	−0.84*	0.89*	0.76*	−0.87*	0.54	0.43
	TN	−0.80*	0.80*	0.78*	−0.45	0.40	0.71*
	CEC	−0.69*	0.90**	0.60	−0.51	0.78*	0.51

*Significant at p < 0.05 and **Significant at p < 0.01.

## 4. Discussion

### 4.1 Influence of Fanya Juu soil and water conservation on the physical properties of soil

The physical properties of soil on agricultural lands are significantly affected by the application of Fanya Juu soil and water conservation practices. In general, as compared to lands with the implementation of Fanya Juu soil and water conservation, lands without Fanya Juu soil and water conservation measures have lower clay and silt content and higher sand content. This is due to unconserved land fine fractions, such as silt and clay, being washed away easily by erosion, leaving heavier and coarser fractions. As a soil conservation structure, the Fanya Juu structure has sediment and runoff trapping action and retains surface runoff and sediments. By doing this, it directly supports soil formation and fertility, which maintains the foundation for agricultural production, water regulation, provisioning service of food and biomass production, thereby securing livelihoods and contributing to climate change adaptation by fostering a more resilient agricultural landscape. In erosion-prone, sloping land, when intensively and continuously cultivated and lacking soil and water conservation measures, it results in the removal of finer particles, and the concentration of coarser sand dominates. The result is in harmony with previous studies [3,19,53], indicating that implementing physical soil and water conservation (SWC) measures shows a lower sand content in comparison to those that do not employ such practices. Besides, literature has documented a higher proportion of clay [54–56] and silt content [26,57] in the lands’ SWC measures in comparison to unconserved lands. On the contrary, some scholars have reported nonsignificant effects on soil texture with the implementation of SWC practices [58,59]. This may be since soil texture is relatively stable in the short to medium term implementation of soil and water conservation practices, and significant texture change may require decades or centuries of sustained management. Besides, in the arid areas, where most of the Fanya Juu structures are used for in situ moisture conservation, the soil textures are dominated by sand [9].

Fanya Juu terraces are designed to decrease the flow velocity of runoff, leading suspended sediments to deposit [3]. The soil aggregate formation is enhanced by accumulated sediments, which results in soil bulk density reduction [60]. Our findings agree with [61] and [62], who reported a decrease in bulk density in Fanya Juu implemented farmlands. The lands with SWC practiced for 5 and 10 years showed higher moisture content; this may be due to the sediment trapping action of these structures, resulting in a reduced flow velocity and encouraging infiltration [63]. The soil’s structural stability index is increased with implementation time and a lower slope position. This may be due to increased soil organic matter accumulation from transported suspended sediments. This is in line with [64], who reported that transporting sediment containing organic matter improved clay and silt content and bulk density in lower slope areas. Besides, silt and clay content increase, and BD reduction may result in enhanced water-holding capacity. The results are consistent with [65] and [54], who reported higher MC in SWC practices than in non-conserved lands.

At the lower slope position, the soil’s physical characteristics improve. This may be due to the accumulation of transported fine soil fractions. This agrees with a report stating that a lower slope has higher clay and silt content and lower BD [66,67]. The reduction in bulk density profoundly enhances the soil physical structure, allowing for deeper root penetration, improved water infiltration, and greater water holding capacity. This translates directly to increased crop resilience during drought, stabilizing production and farm income in an increasingly variable climate. The result of this study points out that the implementation of soil and water conservation on erosion-prone degraded land improves soil physical characteristics.

### 4.2 Influence of Fanya Juu soil and water conservation on chemical properties of soil

From the soil chemical properties studied across age and slopes, a significant influence is observed on land treated with Fanya Juu soil and water conservation. The pH value of land treated with Fanya shows a significant influence on the non-treated land. This result is in agreement with [29,68], who reported a significant influence observed with soil and water conservation implementation on pH. The result is contrary to different scholars who reported that no significant influence on pH is observed with implementation age [69,70]. This may be due to the continued cropping system without sufficient organic matter replenishment, which can lead to soil acidification over time due to depletion of basic cations such as calcium, magnesium, and potassium through crop uptake, the use of acid-forming nitrogen fertilizers, and the local ecological setup, including rainfall patterns, parent material with high buffering capacity [29].

Affecting soil physicochemical and biological activity, soil organic carbon is essential for soil productivity and sustainability, as well as for water and nutrient retention and cycling, and it improves soil structure and aggregate stability. The Fanya Juu soil and water conservation measure, practiced for 10 years in the study area, greatly influenced soil organic carbon. The soil organic carbon in the treated area is significantly higher than that of the non-conserved area. This is due to the retarding action of Fanya Juu on runoff and sediment transportation. Besides that, the effective implementation of soil and water conservation results in higher biomass and residue formation, improving productivity and soil organic carbon management [71]. These fine particles, settled as clay, have a strong influence on soil organic carbon [3,55]. The soil organic carbon increment with the implementation of soil and water conservation measures is reported by different scholars [3,37,59,72,73]. The concentration of soil organic carbon in both treated and non-conserved areas, according to [74], is in the very low range. This is due to intensive tillage, continuous cropping, removal of crop residue, and complete removal of biomass [3]. Since the soil organic carbon is a major terrestrial carbon sink, storing two to three times more carbon than the atmosphere, the low soil organic carbon level implies reduced carbon sequestration. and low nutrient-supplying capacity [37,59]. While Fanya Juu is effective in controlling physical soil loss, our results demonstrate that it is a necessary but insufficient intervention. To break the cycle of organic carbon depletion and achieve resilient agricultural systems, the implementation of Fanya Juu must be systematically integrated with practices that actively replenish organic matter, including cover crops, crop residues, compost, and manure application. [75].

As compared to non-conserved land, higher Available Phosphorus and K^+^ were observed in fields treated with Fanya Juu for 5 and 10 years. This may be attributed to the binding action of nutrients to fine clay micelles [76], and the nutrients associated with increased organic matter contents from the implementation of conservation [28,77]. This finding is in agreement with [34], who reported significantly higher K^+^ and Av. P values with the application of conservation structures in comparison with non-conserved structures. The same results were reported by different stone and soil bunds [27,73,78].

The concentration of TN was observed to be greater in fields utilizing Fanya Juu compared to those lacking soil and water conservation. This discrepancy may be attributed to retaining topsoil and organic matter in fields that employ Fanya Juu soil and water conservation techniques. Besides this, the nitrogen inputs are heightened by planting grass cover and fast-growing legumes to stabilize the terraces [79]. The increase in TN is not merely a result of sediment trapping but the product of a revitalized soil ecosystem. While physical barriers initially concentrate nitrogen-rich organic matter, this creates a foundation for a biological boom: the improved moisture and organic carbon fuel a surge in free-living, nitrogen-fixing bacteria, which convert atmospheric N_2_ into bioavailable forms, creating an in-situ bio-fertilizer factory. Similar results are reported in the Fanya Juu soil and water conservation to improve soil total nitrogen concentrations [31,32,57,80]. In contrast to this finding, the TN content did not show a significant difference in lands treated with soil and water conservation measures [81]. The discrepancy between our findings and those [29], regarding TN content under soil and water conservation measures, could be attributed to differences in climate, soil properties, and land use management

With implementation age, a significant increment in CEC was observed in both 5- and 10-year soil and water conservation practices than in non-conserved lands. This may be due to the accumulation of soil organic matter and clay content in Fanya Juu structures. The result is in agreement with some scholars who reported a significant influence on CEC with the implementation of SWC [82,83]. Reports by [19,73] showed differences in CEC, found to be insignificant in conserved and non-conserved areas. The measured improvements directly catalyze the socio-economic benefits essential for sustainable food systems. The 39% increase in CEC means a smallholder’s fertilizer applications are significantly more effective, reducing costs and environmental leaching while increasing nutrient availability for higher yields.

### 4.3 The heterogeneity of soil properties among various slope classes

The soil sand content is higher in the up-slope position, which may be ascribed to the transport of fine soil particles due to erosion occurring in the upper part slope. This resulted in the subsequent accumulation of silt and clay content, which is significantly higher in deposition areas. The result agrees with reports by [32,68,84] and [85], who found that higher accumulation of clay and organic matter washed down from up slopes left a coarser fraction. The bulk density of soils is higher in the upslope position. This may be attributed to the washing away of fine soil fractions and organic carbon. The Soil Structural Stability Index (SSSI) serves as a significant metric for assessing soil quality and health, given that structural integrity and stability of soil are essential for sustaining the fertility of the soil, facilitating water infiltration, and enhancing resistance to erosion. The land treated with soil and water conservation in the lower slope showed significantly higher SSSI in the upslope and non-treated lands.

This is consistent with a report showing low structural damage in soils of depositional areas [36]. The soil moisture content and organic carbon showed significantly higher values in lower slope positions due to the accumulation of fine fractions and organic matter, improving the water retention capacity of soils. A significant difference was observed in lower deposition areas than in up-slopes regarding the pH of the soil. Similar reports are obtained by [86], stating increased pH in the deposition area. Soil organic matter and total nitrogen are significantly higher in deposition zones than in up-slope loss and transportation zones [3,68,87,88]. The available phosphorus did not show a significant difference, and exchangeable potassium showed a significant difference in the slope position. This is because clay and organic matter content affect the availability of potassium [89].

The correlation analysis demonstrated significant associations between soil characteristics across age classes and slope positions. Specifically, silt and clay fractions exhibited strong positive correlations with pH, organic carbon, total nitrogen, available phosphorus, and cation exchange capacity, suggesting that soils with finer textures enhance nutrient retention and overall fertility. In contrast, sand content was negatively correlated with most chemical parameters, indicating that coarser soils have a diminished capacity to retain nutrients and moisture. Soil moisture content and aggregate stability showed a positive relationship with fertility indicators, reflecting improved soil structure and increased biological activity under conditions of increased moisture and well-developed aggregation. Conversely, bulk density was negatively correlated with organic carbon, total nitrogen, and exchangeable potassium, implying that greater soil compaction adversely affects organic matter accumulation and nutrient availability. This is in agreement with reports from scholars stating positive and negative correlations with soil physical characteristics [25,72].

The two-way ANOVA analysis is the consistent and statistically significant interaction effect (Age* Slope) observed across nearly all measured soil properties. While the main effects of Age classes and slope classes are themselves highly significant(p < 0.00), the presence of significant interaction terms (p < 0,05) for silt, clay BD, MC, OC, TN, PH, Av. P, K^+^, and CEC) reveals a more complex, non-additive relationship. This indicates the influence of land slope on a given soil property is not uniform but is instead dependent on the age class, and conversely, the effect of age varies significantly across different slope gradients. For instance, the relationship between slope and soil organic carbon is not a simple, standalone effect but is fundamentally modified by the age of the Fanya Juu. This study shows that the evolution of soil characteristics is governed by the synergistic interplay between topographic position and implementation age.

## Conclusions

The study intended to pinpoint the significant influence of Fanya Juu soil and water conservation structure on soil physicochemical characteristics in restoring and improving erosion-prone, degraded, sloping agricultural lands under high population pressure. The key findings revealed that implementing Fanya Juu SWC significantly lowers the sand content and BD of soil. Whereas silt, clay, MC, SSSI, pH, SOC, and Av. P, K^+^, and TN, CEC increased with implementation. The age of the terraces is a crucial aspect, since 10-year-old Fanya Juu terraces exhibit notable enhancements in SOC, available Phosphorus, total nitrogen, moisture content, and aggregate stability, particularly in the upper and intermediate slope categories.

This research uniquely investigates the impact of Fanya Juu implementation age and slope position on soil health and quality in degraded and dry areas, in combating nutrient deficiency for production crops resulting from soil erosion. These findings have substantial implications for agricultural professionals and policymakers prioritizing sustainable land management and livelihoods.

The Ministry of Agriculture, the Sustainable Land Management (SLM) Program, and regional agricultural bureaus should prioritize Fanya Juu terraces as a central component of land rehabilitation and soil conservation policies, particularly in erosion-prone and densely populated highland areas. For large-scale adoption, the integration of the Productive Safety Net Program (PSNP) and the community-based watershed management project is recommended. Since older terraces exhibit greater soil quality enhancements, local governments and extension offices should establish terrace maintenance schedules and community-based monitoring protocols. To enhance climate resilience, Fanya Juu should be integrated with climate-smart and agroforestry practices; for instance, planting deep-rooted, drought-tolerant crops or nitrogen-fixing trees along terrace bunds. Incorporating organic amendments (compost, manure) can further improve soil structure, aggregate stability, and water retention to address climate change resilience.

This study has several limitations. The chronosequence approach assumes site similarity before treatment, which cannot be fully verified in the absence of pre-implementation data, and all the measurements were collected within a single climatic year, making the results potentially sensitive to interannual variability. As a single-site study, the broader applicability of findings remains uncertain. Besides these limitations, the absence of crop yield data, economic analysis, or hydrological impacts has not been incorporated. Future research should move beyond soil fertility assessment to include cost-benefit analysis; long-term monitoring and multi-site studies are required, inclusion of crop yield responses, and climate adaptation potential of Fanya Juu implementation and traditional farming, as well as the adaptation potential of Fanya Juu under extreme weather conditions.

Collaboration between research institutions and extension services should focus on developing monitoring frameworks to quantify both economic and ecological outcomes. Such investigations will elucidate the potential of these environmental policies to facilitate sustainable development
